# Shorter Anogenital Distance in Women with Adenomyosis Diagnosed by MUSA 2022 Criteria: A Prospective Case–Control Study

**DOI:** 10.3390/jcm15041319

**Published:** 2026-02-07

**Authors:** Berivan Guzelbag, Aysegul Bestel, Sevim Ezgi Katran, Betul Averbek, Hale Goksever Celik

**Affiliations:** 1 Department of Obstetrics and Gynecology, University of Health Sciences, Haseki Training and Research Hospital, 34270 Istanbul, Türkiye; 2 Department of Obstetrics and Gynecology, University of Health Sciences, Kanuni Sultan Suleyman Training and Research Hospital, 34303 Istanbul, Türkiye; draysegulciftci@gmail.com; 3 Department of Obstetrics and Gynecology, University of Health Sciences, Sisli Hamidiye Etfal Training and Research Hospital, 34371 Istanbul, Türkiye; katranezgi5@gmail.com; 4 Department of Obstetrics and Gynecology, Liv Hospital Bahcesehir, Istinye University, 34517 Istanbul, Türkiye; betul_averbek@hotmail.com; 5 Department of Obstetrics and Gynecology, Acibadem University, 34638 Istanbul, Türkiye; hgoksever@yahoo.com; 6 IVF and Endometriosis Center, Acibadem Fulya Hospital, 34349 Istanbul, Türkiye

**Keywords:** adenomyosis, anogenital distance, transvaginal ultrasound, estrogen-dependent disorders, non-invasive diagnostic marker

## Abstract

**Objective:** The objective was to investigate the association between anogenital distance (AGD) and adenomyosis in reproductive-age women and to evaluate the potential of AGD as a non-invasive biomarker reflecting prenatal hormonal environment. **Methods:** This prospective case–control study included 40 women with adenomyosis diagnosed according to the Morphological Uterus Sonographic Assessment (MUSA) 2022 criteria and 40 age-matched healthy controls. Two AGD measurements were obtained: AGD-af (anus to posterior fourchette) and AGD-act (anus to clitoral tip). Measurements were performed by two independent observers using vernier calipers. Hormonal parameters, reliability analyses, receiver operating characteristic (ROC) curve analysis, and logistic regression were conducted. **Results:** Women with adenomyosis had significantly shorter AGD-af compared to controls (23.78 ± 7.20 vs. 27.88 ± 7.50 mm, *p* = 0.015), whereas AGD-act did not differ significantly (*p* = 0.574). Inter- and intra-observer reliability was excellent (intraclass correlation coefficient [ICC] = 0.87–0.93). ROC analysis revealed an area under the curve (AUC) of 0.658 (95% confidence interval [CI]: 0.55–0.76) for AGD-af (optimal cut-off = 24 mm; sensitivity: 57.5%, specificity: 67.5%). In multivariate logistic regression, AGD-af remained independently associated with adenomyosis after adjusting for age and body mass index (BMI) (adjusted odds ratio [OR] = 0.925, 95% CI = 0.866–0.989, *p* = 0.022). No significant difference was observed in hormonal parameters between groups. **Conclusions**: Women with adenomyosis exhibit a modest but significant reduction in AGD-af, suggesting a possible influence of prenatal hormonal environment in disease pathogenesis. Although its diagnostic accuracy is fair, AGD-af may serve as a complementary, non-invasive biomarker in clinical assessment of adenomyosis.

## 1. Introduction

Adenomyosis involves the ectopic infiltration of endometrial glands and stromal cells into the uterine muscular wall, often accompanied by reactive myometrial hypertrophy [1]. Prevalence estimates differ considerably across studies (5–70%), largely reflecting heterogeneity in diagnostic approaches and cohort selection; among subfertile women, a pooled prevalence of roughly 10% has been documented [2,3]. The condition manifests clinically with dysmenorrhea, heavy or irregular menstrual bleeding, persistent pelvic pain, and impaired fertility, all of which compromise both quality of life and reproductive potential [4,5,6]. Although approximately one-third of affected women may be asymptomatic, adenomyosis frequently coexists with other estrogen-dependent conditions, particularly endometriosis and uterine leiomyomas [1,6]. Furthermore, meta-analyses have demonstrated that adenomyosis is associated with reduced live birth rates and increased miscarriage rates in women undergoing assisted reproductive technologies [7]. Growing awareness that adenomyosis also affects women of reproductive age, with demonstrable adverse effects on fertility, underscores the importance of timely and precise identification.

Until recently, confirming adenomyosis depended on histological analysis of surgically removed uteri, restricting diagnosis to patients no longer desiring pregnancy [1]. Advances in transvaginal ultrasound (TVUS) and magnetic resonance imaging (MRI) have since made non-invasive detection feasible, with reported sensitivity of 72–82% and specificity of 78–85% [8,9]. The Morphological Uterus Sonographic Assessment (MUSA) consensus subsequently established uniform sonographic criteria for adenomyosis, enhancing inter-center diagnostic consistency [10]. Nevertheless, recognizing adenomyosis at an early stage remains difficult, highlighting the demand for supplementary non-invasive markers capable of identifying at-risk individuals before overt disease develops.

Anogenital distance (AGD) refers to the span measured from the anal center to specific perineal landmarks: the scrotal base (AGD-as) or penile insertion (AGD-ap) in males, and the posterior fourchette (AGD-af) or clitoris (AGD-ac) in females [11,12,13]. This measurement displays marked sexual dimorphism—roughly twofold longer in males than females—and is shaped by fetal androgen levels during the 8–14-week masculinization programming window [14]. In postnatal life, AGD serves as a validated surrogate of in utero androgen exposure that remains largely constant after adjusting for body habitus [13]. These properties position AGD as a promising non-invasive tool for probing the prenatal hormonal milieu and its links to diseases manifesting in adulthood.

AGD has been increasingly recognized as a non-invasive marker associated with a wide range of reproductive disorders in both sexes, including cryptorchidism, hypospadias, and reduced semen quality in males, and endometriosis, polycystic ovary syndrome, and subfertility in females [15]. Accumulating evidence links AGD to prevalent gynecological conditions, reinforcing the notion that the prenatal hormonal milieu shapes susceptibility to adult-onset disease. Endometriosis has been repeatedly associated with reduced AGD relative to healthy women, implying diminished prenatal androgen exposure [16,17], whereas women with polycystic ovary syndrome (PCOS)—a hyperandrogenic phenotype—tend to display longer AGD values [18,19]. A systematic review synthesizing these observations concluded that endometriosis and PCOS may occupy opposite ends of the prenatal androgen spectrum [20]. Because adenomyosis and endometriosis share key pathophysiological features—notably estrogen-dependence and overlapping symptom profiles—it is biologically plausible that adenomyosis is likewise associated with altered AGD.

Despite the growing evidence linking AGD to gynecological disorders, the relationship between AGD and adenomyosis remains largely unexplored. Adenomyosis and endometriosis share an estrogen-driven pathophysiology in which localized estrogen excess and diminished progesterone responsiveness are key mechanistic drivers [21]. Thus far, a single retrospective analysis has examined this association, observing significantly reduced AGD-af in adenomyosis patients relative to unaffected controls [22]. However, no prospective data exist on AGD in women whose adenomyosis was confirmed through standardized sonographic criteria. Accordingly, we aimed to compare AGD measurements between women with MUSA 2022-defined adenomyosis and healthy controls. Our hypothesis was that adenomyosis would be accompanied by shorter AGD, mirroring the pattern documented in endometriosis and pointing toward a shared prenatal hormonal origin.

## 2. Methods

We conducted a prospective case–control investigation between May and August 2023 in the Obstetrics and Gynecology Department of Kanuni Sultan Suleyman Training and Research Hospital (Istanbul Health Sciences University, Istanbul, Türkiye). Ethical clearance was granted by the institutional Clinical Research Ethics Committee (Decision No: 40, Date: 12 April 2023), and every participant provided written informed consent. Eligible participants were non-pregnant women aged 30–45 years. The adenomyosis group (*n* = 40) comprised patients whose diagnosis was established via transvaginal ultrasonography following MUSA 2022 criteria. The 2022 MUSA consensus defines and classifies ultrasonographic features but does not specify minimum diagnostic criteria. For this study, adenomyosis diagnosis required identification of at least one direct sonographic feature (myometrial cysts, hyperechoic islands, or echogenic subendometrial lines and buds) plus a minimum total of four direct and indirect features (asymmetrical myometrial wall thickening, globular uterus, irregular or interrupted junctional zone, fan-shaped shadowing, or translesional vascularity). All ultrasonographic examinations were performed by an experienced gynecologist with expertise in gynecological ultrasound using a Voluson E8 ultrasound system (GE Healthcare, Chicago, IL, USA). Controls (*n* = 40) were age-matched women attending routine gynecological visits whose clinical and ultrasonographic assessments revealed no pelvic pathology. Endometriosis was ruled out through a combined evaluation of medical history, transvaginal sonography, and bimanual pelvic examination. PCOS was screened for using the revised 2023 Rotterdam criteria. Exclusion criteria for both groups encompassed concurrent endometriosis, PCOS, metabolic comorbidities (diabetes mellitus, thyroid disorders), pelvic organ prolapse, previous oncological therapy, and any exposure to hormonal contraceptives, ovulation induction agents, or anti-androgen drugs within the preceding three months.

To limit the influence of cyclical hormonal variation, all evaluations—including venipuncture and AGD assessment—were scheduled in the early follicular phase (cycle days 2–5) to minimize potential variability related to hormonal fluctuations throughout the menstrual cycle. The hormonal panel comprised total testosterone, free androgen index (FAI), androstenedione, dehydroepiandrosterone sulfate (DHEAS), sex hormone-binding globulin (SHBG), 17-hydroxyprogesterone (17-OHP), and prolactin. Clinical signs of androgen excess were quantified with the modified Ferriman–Gallwey (mFG) scoring system. These hormonal and clinical data, together with transvaginal ultrasonographic findings and menstrual history, enabled systematic ruling out of PCOS per the 2023 Rotterdam criteria, as well as non-classic congenital adrenal hyperplasia and hyperprolactinemia.

**Figure 1:**
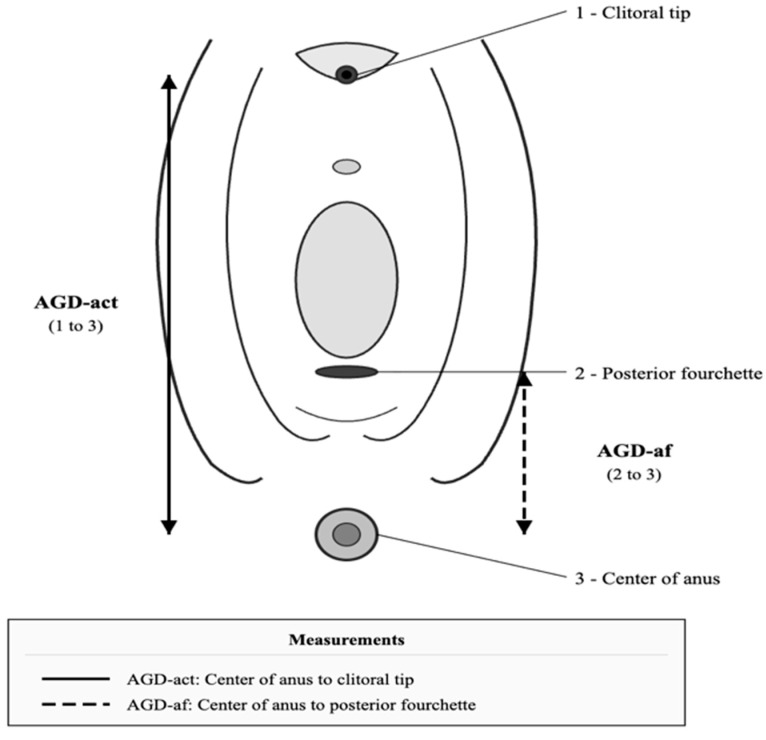
Schematic illustration demonstrating landmarks for anogenital distance (AGD) measurements. 1: clitoral tip; 2: posterior fourchette; 3: center of anus. AGD-act (solid line): distance from the center of the anus to the clitoral tip (points 3 to 1). AGD-af (dashed line): distance from the center of the anus to the posterior fourchette (points 3 to 2).

Anogenital distance measurements were performed using a Vernier caliper (INSIZE Co., Ltd., Suzhou, China). Measurements were taken with women in the lithotomy position with thighs at a 45-degree angle. Two experienced gynecologists independently measured each distance twice. Two AGD measurements were obtained: AGD-act (from the center of the anus to the clitoral tip) and AGD-af (from the center of the anus to the posterior fourchette) (Figure 1). The average values of all measurements were recorded in millimeters.

### 2.1. Statistical Analysis

The required sample size was derived from published AGD data in an endometriosis cohort sharing comparable estrogen-driven pathophysiology [23]. Setting α at 0.05 and statistical power at 80% yielded a minimum requirement of 23 participants per arm; enrollment was extended to 40 per group. All analyses were carried out in SPSS v.26.0 (IBM Corp., Armonk, NY, USA). Distribution normality was evaluated with the Shapiro–Wilk test; given the non-Gaussian distribution observed, non-parametric methods were adopted. Continuous data are reported as mean ± SD alongside median and interquartile range; categorical data are presented as counts and proportions. Between-group differences in continuous variables were assessed with the Mann–Whitney U test, and categorical comparisons employed the chi-square or Fisher’s exact test where appropriate. Relationships between AGD-af and anthropometric variables (age, BMI) were examined with Pearson correlation coefficients, justified by the sufficient sample size and the robustness of this test to mild departures from normality; Spearman rank correlation served as a confirmatory analysis. Diagnostic accuracy of AGD-af was evaluated through receiver operating characteristic (ROC) analysis; the area under the curve (AUC) was computed and the optimal threshold identified by maximizing the Youden index (sensitivity + specificity − 1). Logistic regression—first univariate, then multivariate—was used to identify independent predictors of adenomyosis. Age and BMI were entered into the multivariable model together with AGD-af; effect sizes are expressed as odds ratios (OR) with 95% confidence intervals (CI). Statistical significance was set at a two-sided *p* < 0.05. Measurement reproducibility was quantified with intraclass correlation coefficients (ICC), computed under a two-way mixed-effects model (absolute agreement, single measures).

### 2.2. Use of Artificial Intelligence

Artificial intelligence tools provided assistance during the language editing and formatting process of this manuscript. ChatGPT Plus (version 5.0) was employed for grammar checking and enhancing clarity of expression. All scientific content, data analysis, result interpretation, and conclusions represent the original work of the authors. Artificial intelligence was not utilized in data collection, statistical analysis, or scientific content generation processes.

## 3. Results

Baseline demographic, clinical, and hormonal data appear in Table 1. The two groups did not differ significantly in age (*p* = 0.726), height (*p* = 0.505), body weight (*p* = 0.255), BMI (*p* = 0.127), gravidity (*p* = 0.855), or parity (*p* = 0.470). Delivery mode (*p* = 0.389), episiotomy history (*p* = 0.356), prior uterine curettage (*p* = 0.223), and smoking status (*p* = 0.650) were also comparable between groups. Every hormonal parameter—total testosterone, androstenedione, DHEAS, SHBG, free androgen index, 17-OHP, and prolactin—fell within normal reference limits, with no inter-group differences detected (all *p* > 0.05). Modified Ferriman–Gallwey scores were similarly matched (2.52 ± 1.75 vs. 2.33 ± 1.19, *p* = 0.687).

**Figure 2:**
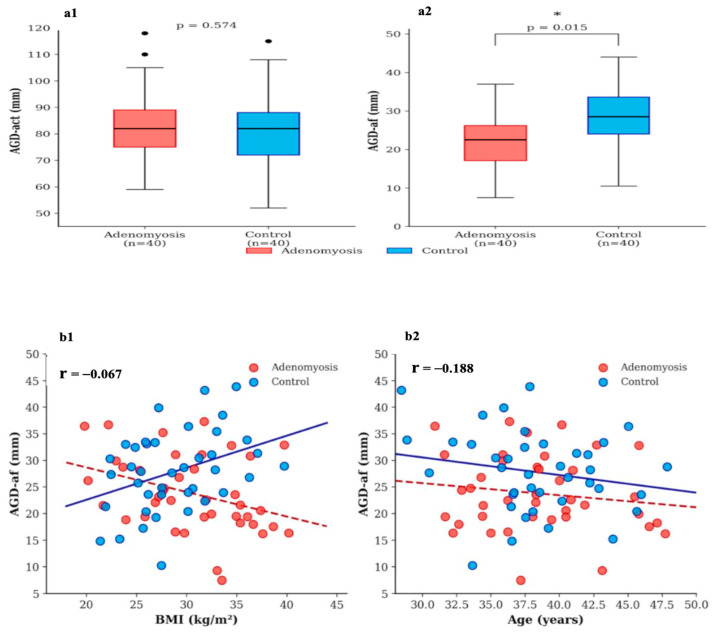
Anogenital distance measurements and correlations. Upper panels: Comparison of AGD measurements between adenomyosis and control groups. (**a1**) AGD-act: distance from the center of the anus to the clitoral tip. (**a2**) AGD-af: distance from the center of the anus to the posterior fourchette. Red boxes represent adenomyosis group, blue boxes represent control group. The horizontal line within each box represents the median, box boundaries represent the interquartile range (25th–75th percentile), and whiskers extend to minimum and maximum values. * *p* < 0.05. Lower panels: Correlation between AGD-af and BMI (**b1**) and age (**b2**). Red circles represent women with adenomyosis, blue circles represent controls. Dashed line: adenomyosis group regression; solid line: control group regression. r = Pearson correlation coefficient.

Measurement reliability was excellent for both AGD-af (intra-observer ICC = 0.91; inter-observer ICC = 0.87) and AGD-act (intra-observer ICC = 0.93; inter-observer ICC = 0.89). Individual AGD values are detailed in Table 2 and Figure 2a. AGD-act did not differ between the adenomyosis and control groups (82.00 ± 12.00 vs. 80.50 ± 12.80 mm, *p* = 0.574). By contrast, AGD-af was significantly reduced in the adenomyosis group relative to controls (23.78 ± 7.20 vs. 27.88 ± 7.50 mm, *p* = 0.015), with a medium effect size (Cohen’s d = 0.56). Neither BMI (r = −0.067, *p* = 0.556) nor age (r = −0.188, *p* = 0.095) correlated significantly with AGD-af; Spearman analysis confirmed these null findings (Figure 2b).

**Figure 3:**
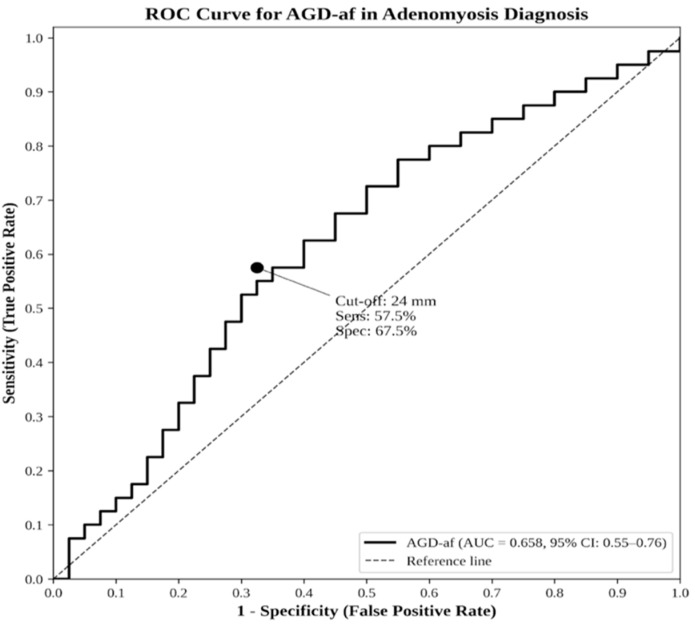
Receiver operating characteristic (ROC) curve for AGD-af in the diagnosis of adenomyosis. AUC = 0.658 (95% CI: 0.55–0.76). The optimal cut-off point (24 mm) determined by the Youden index is indicated.

Receiver operating characteristic (ROC) curve analysis was performed to evaluate the diagnostic performance of AGD-af for adenomyosis (Figure 3). The area under the curve (AUC) was 0.658 (95% CI: 0.55–0.76), indicating fair discriminatory ability. Using the Youden index, the optimal cut-off value was determined as 24 mm, yielding a sensitivity of 57.5% and specificity of 67.5%.

Logistic regression analysis was performed to evaluate the association between AGD-af and adenomyosis (Table 3). In univariate analysis, AGD-af was significantly associated with adenomyosis (OR: 0.926, 95% CI: 0.868–0.987, *p* = 0.019). After adjusting for age and BMI in the multivariate model, AGD-af remained an independent predictor of adenomyosis (adjusted OR: 0.925, 95% CI: 0.866–0.989, *p* = 0.022). Each 1 mm increase in AGD-af was associated with a 7.5% decrease in the odds of having adenomyosis.

## 4. Discussion

This study investigated the relationship between anogenital distance and adenomyosis in reproductive-age women. Our main finding is that women with adenomyosis have significantly shorter AGD-af compared to controls (23.78 ± 7.20 vs. 27.88 ± 7.50 mm, *p* = 0.015), whereas AGD-act did not differ between groups. To the best of our knowledge, this is the first prospective case–control study to examine AGD measurements in women with adenomyosis diagnosed using standardized MUSA 2022 criteria [10]. Our findings support the hypothesis that prenatal hormonal environment may play a role in the pathogenesis of adenomyosis, consistent with the recent report by Liu et al. [22], who independently demonstrated a similar association between shorter AGD-af and adenomyosis.

In the present study, adenomyosis was diagnosed using ultrasonographic assessment based on the MUSA 2022 criteria without histopathological confirmation. Although histopathological examination of hysterectomy specimens remains the gold standard for definitive diagnosis, this approach is inherently limited to women undergoing hysterectomy and is therefore not feasible in the majority of clinical and research settings. Importantly, transvaginal ultrasound has demonstrated reliable diagnostic performance for adenomyosis. A structured meta-analysis by Tellum et al. reported pooled sensitivity and specificity of 78% for ultrasound diagnosis of adenomyosis using histopathology as the reference standard [9]. More recently, Yavuz et al. evaluated the revised MUSA 2022 features in patients with histopathologically confirmed adenomyosis and demonstrated that a combination of more than four sonographic features was a significant predictor, with the combination of three direct and two or more indirect features achieving 100% specificity [24]. Furthermore, Hu et al. developed a MUSA-based ultrasound scoring system that achieved an AUC of 0.894, with sensitivity of 79.4% and specificity of 83.9% in a validation cohort [25]. These findings support the use of the MUSA 2022 criteria as a reliable non-invasive diagnostic framework for adenomyosis in clinical research settings.

Our finding that AGD-af, but not AGD-act, was significantly shorter in women with adenomyosis is consistent with the pattern observed in endometriosis studies. Mendiola et al. first reported that shorter AGD-af was strongly associated with endometriomas and deep infiltrating endometriosis [17]. Crestani et al. confirmed this in a prospective study with laparoscopic and histological verification, reporting AGD-af values of 21.5 mm in women with endometriosis versus 32.3 mm in controls [16]. Similar findings were reported using MRI-based measurements [26,27]. A systematic review by Pan et al. concluded that shorter AGD-af is consistently associated with endometriosis [20]. The similarity between our adenomyosis findings and endometriosis literature is noteworthy, given that both conditions share estrogen-dependent pathophysiology and frequently coexist [1,28,29,30]. The differential pattern between AGD-af and AGD-act can be explained by anatomical factors; the posterior fourchette provides a more stable landmark compared to the anterior clitoral surface, which is more susceptible to variability due to subcutaneous fat distribution [11,13]. Liu et al. similarly reported that only AGD-af was significantly reduced in adenomyosis patients, suggesting that AGD-af may be more suitable as a clinical biomarker [22]. Notably, our finding that AGD-af, rather than AGD-act, was significantly associated with adenomyosis is consistent with recent evidence suggesting that AGD-af is a more reliable measurement for assessing prenatal hormonal exposure. Liu et al. demonstrated that AGD-af is insensitive to obstetric factors such as vaginal delivery and episiotomy (*p* = 0.88), whereas AGD-ac may be affected by these factors [22]. This suggests that AGD-af better reflects the original prenatal hormonal environment without being confounded by postnatal obstetric events. Furthermore, multiple studies have confirmed that the association between AGD-af and estrogen-dependent gynecological conditions remains significant even after adjusting for vaginal delivery and episiotomy [16,20,22]. Our results align with these observations and support the use of AGD-af as the preferred measurement in studies investigating associations between AGD and gynecological disorders.

The link between reduced AGD and adenomyosis can be interpreted through the lens of prenatal hormonal programming. AGD is determined within the 8–14-week gestational window of sexual differentiation, capturing the equilibrium between fetal androgen and estrogen signaling [14,31]. Relatively lower androgen or elevated estrogen concentrations during this critical interval yield shorter AGD in females [32]. Adenomyosis is widely recognized as an estrogen-driven disorder, marked by heightened local aromatase expression and dysregulated estrogen metabolism [33]. Experimental animal data show that perinatal contact with estrogenic agents—notably diethylstilbestrol and bisphenol A—raises the likelihood of adult-onset adenomyosis [34,35,36]. This contrasts with the longer AGD observed in hyperandrogenic PCOS, reinforcing the view that AGD mirrors the prenatal hormonal balance [19]. Notably, the AGD–PCOS association is stronger, presumably because androgen excess directly influences AGD [20]. Adenomyosis, by contrast, is driven primarily by estrogen dominance and progesterone resistance rather than androgen surplus [29], potentially accounting for the more modest association in our cohort. This hormonal distinction implies that, although both conditions trace back to the prenatal hormonal environment, their underlying mechanisms diverge considerably. Taken together, these observations support the notion that women who later develop adenomyosis may have experienced a relatively hyperestrogenic or hypoandrogenic intrauterine environment, manifested as shorter AGD-af and consistent with the molecular pathways implicated in adenomyosis [37,38]. It should be noted that there are currently no validated serum biomarkers in adult women that can retrospectively reflect prenatal hormonal exposure. AGD remains the most reliable postnatal anthropometric marker of the fetal hormonal environment [13,14].

In our study, ROC analysis revealed that AGD-af had a fair discriminatory ability for adenomyosis with an AUC of 0.658. Using a cut-off value of 24 mm, the sensitivity and specificity were 57.5% and 67.5%, respectively. These values are lower than those reported for endometriosis by Crestani et al., who found an AUC of 0.840, and Sánchez-Ferrer et al., who reported an AUC of 0.91 for deep infiltrating endometriosis. Sánchez-Ferrer et al. demonstrated that combining AGD with anti-Müllerian hormone (AMH) improved diagnostic accuracy [16,23,39]. The fair diagnostic accuracy suggests that AGD-af alone may not be sufficient as a standalone marker for adenomyosis; however, it could serve as a complementary non-invasive parameter in initial clinical assessment. The developmental origins of adenomyosis have been increasingly recognized, with immunological alterations also playing a role in disease pathogenesis [40,41,42,43]. Additionally, AGD measurements did not correlate with age or BMI in either group, which is consistent with previous reports suggesting that AGD is primarily determined during the prenatal period [44,45,46].

From a clinical perspective, our findings suggest that AGD-af may represent a potential non-invasive anthropometric parameter associated with adenomyosis risk, although its modest diagnostic accuracy (AUC 0.658) precludes its use as a standalone screening tool. Nevertheless, these preliminary findings may encourage further investigation into whether AGD-af could serve as a complementary parameter within multimodal diagnostic approaches that integrate clinical symptoms, anthropometric measurements, and imaging findings. Given the shared prenatal hormonal etiology between adenomyosis and endometriosis, future research should also explore whether AGD-af has utility as a common marker for estrogen-dependent gynecological conditions.

Future research should prioritize longitudinal birth cohort studies measuring maternal and cord blood hormone levels with long-term gynecological follow-up to directly test the prenatal programming hypothesis in adenomyosis. Additionally, the development of multiparametric diagnostic models integrating anthropometric, ultrasonographic, and biochemical parameters warrants investigation in larger multicenter cohorts.

This work has several notable strengths. It represents the first prospectively designed investigation targeting the AGD–adenomyosis relationship within a thoroughly characterized cohort. A prospective case–control framework with standardized diagnostics was employed; adenomyosis was identified according to MUSA 2022 consensus criteria, offering a reproducible and internationally recognized classification [10]. AGD was measured independently by two experienced gynecologists who achieved excellent reproducibility (ICC = 0.87–0.93), satisfying ‘good to excellent’ reliability thresholds [47]. Furthermore, controls were closely matched for age and BMI, reducing confounding potential, and concurrent endometriosis was actively assessed and excluded, ensuring that AGD differences could be attributed specifically to adenomyosis. Additionally, the single-center design ensured uniform diagnostic protocols and consistent application of strict exclusion criteria, including systematic exclusion of endometriosis, PCOS, and hormonal medication use, throughout the study.

Several limitations merit acknowledgment. The observational design inherently prevents causal inference regarding the link between reduced AGD and adenomyosis. Longitudinal studies following women from birth would be needed to confirm the prenatal programming hypothesis. Second, although TVUS using MUSA 2022 criteria has demonstrated high diagnostic accuracy for adenomyosis, histopathological confirmation was not performed, as it requires hysterectomy specimens, which was not applicable since none of the participants had undergone hysterectomy. However, MUSA 2022 criteria have shown sensitivity of 79–89% and specificity of 67–98% for ultrasound-based diagnosis of adenomyosis [9,10]. Third, this single-center study with a relatively small sample size may limit generalizability. The sample size was constrained by the short recruitment period (May–August 2023), strict exclusion criteria were applied to minimize confounding, and the limited number of patients meeting MUSA 2022 criteria within the study period. However, the sample size exceeded the minimum required by our a priori power analysis. Future multicenter studies with larger and ethnically diverse populations are warranted to validate these findings. Fourth, the potential confounding effect of obstetric history on AGD measurements warrants consideration. Future studies focusing exclusively on nulliparous women would provide more definitive evidence. Fifth, although smoking status was recorded and did not differ between groups, detailed data on smoking duration (pack-years) and alcohol consumption were not collected, which may represent unmeasured confounders. However, alcohol consumption is generally low among reproductive-age women in Türkiye due to sociocultural factors. Future studies should incorporate these variables into regression models to better adjust for their potential impact on AGD measurements. Sixth, the current sample size did not allow for meaningful stratification of adenomyosis subtypes (e.g., focal vs. diffuse). Future studies with larger cohorts should investigate whether AGD differs across adenomyosis subtypes, which may provide further insight into the pathogenic mechanisms underlying different forms of the disease.

## 5. Conclusions

In conclusion, the present prospective investigation reveals a significant reduction in AGD-af among women with adenomyosis relative to healthy controls, lending support to the premise that the prenatal hormonal milieu may participate in adenomyosis pathogenesis. Although AGD-af demonstrates only fair discriminatory power, it holds promise as a complementary non-invasive biomarker in clinical settings. Future efforts should prioritize: (1) multicenter validation of the proposed AGD-af threshold across diverse populations, (2) longitudinal cohorts to determine whether AGD can anticipate adenomyosis onset prior to symptom emergence, (3) exploration of AGD variation among adenomyosis subtypes (focal vs. diffuse), and (4) development of composite diagnostic panels integrating AGD with imaging and serum biomarkers.

## Figures and Tables

**Table 1:** Demographic, clinical and hormonal characteristics of study participants.

Variable	Adenomyosis (*n* = 40)	Control (*n* = 40)	*p*-Value
**Age (years)**			0.726
Mean ± SD	38.45 ± 4.62	38.02 ± 4.89	
Median (range)	38.5 (30–45)	38.0 (30–45)	
**Height (m)**			0.505
Mean ± SD	1.60 ± 0.06	1.60 ± 0.05	
Median (range)	1.60 (1.50–1.70)	1.60 (1.50–1.70)	
**Body weight (kg)**			0.255
Mean ± SD	77.53 ± 11.96	74.38 ± 12.54	
Median (range)	80.0 (58–105)	72.0 (55–100)	
**BMI (kg/m^2^)**			0.127
Mean ± SD	30.56 ± 5.54	28.81 ± 4.57	
Median (range)	29.8 (21.2–41.0)	28.3 (21.0–39.1)	
**Gravidity**			0.855
Mean ± SD	3.20 ± 2.03	3.12 ± 1.62	
Median (range)	3 (0–7)	3 (0–6)	
**Parity**			0.470
Mean ± SD	2.35 ± 1.33	2.15 ± 1.12	
Median (range)	2 (0–4)	2 (0–4)	
**Delivery mode**			0.389
**Vaginal only**	12 (30.0%)	10 (25.0%)	
**Cesarean only**	17 (42.5%)	18 (45.0%)	
**Both (vaginal + cesarean)**	6 (15.0%)	4 (10.0%)	
**Nulliparous**	5 (12.5%)	8 (20.0%)	
**Episiotomy (+)**	18 (45.0%)	14 (35.0%)	0.356
**3rd–4th-degree perineal tear**	0 (0%)	0 (0%)	—
**History of uterine curettage**			0.223
Yes (+)	15 (37.5%)	9 (22.5%)	
Total number, Mean ± SD	0.75 ± 1.32	0.35 ± 0.70	0.141
**Smoking (+)**	15 (37.5%)	18 (45.0%)	0.650
**Hormonal parameters**			
Total Testosterone (ng/mL)	0.18 ± 0.10	0.21 ± 0.11	0.206
Androstenedione (ng/mL)	1.80 ± 0.68	2.03 ± 0.60	0.079
DHEAS (μg/dL)	168.50 ± 75.80	170.20 ± 78.40	0.922
SHBG (nmol/L)	61.40 ± 30.20	56.80 ± 28.50	0.486
Free Androgen Index	0.38 ± 0.32	0.45 ± 0.36	0.361
17-OHP (ng/mL)	0.58 ± 0.18	0.66 ± 0.23	0.116
Prolactin (ng/mL)	15.20 ± 8.80	14.90 ± 8.50	0.877
mFG Score	2.52 ± 1.75	2.33 ± 1.19	0.687

Continuous variables were compared using the Mann–Whitney U test. Categorical variables were compared using the chi-square test or Fisher’s exact test, as appropriate. 17-OHP: 17-hydroxyprogesterone; DHEAS: dehydroepiandrosterone sulfate; SHBG: sex hormone-binding globulin; mFG: modified Ferriman–Gallwey.

**Table 2:** Anogenital distance measurements of study participants.

Variable	Adenomyosis (*n* = 40)	Control (*n* = 40)	*p*-Value
AGD-act (mm)			0.574
Mean ± SD	82.00 ± 12.00	80.50 ± 12.80	
Median (IQR)	82.0 (75.0–89.0)	82.0 (72.0–88.0)	
AGD-af (mm)			0.015
Mean ± SD	23.78 ± 7.20	27.88 ± 7.50	
Median (IQR)	22.5 (17.1–26.2)	28.5 (24.0–33.6)	

IQR: Interquartile range (25th–75th percentile). Groups were compared using the Mann–Whitney U test.

**Table 3:** Logistic Regression Analysis for Adenomyosis.

Variable	Univariate OR (95% CI)	*p*	Adjusted OR (95% CI)	*p*
AGD-af (mm)	0.926 (0.868–0.987)	0.019 *	0.925 (0.866–0.989)	0.022 *
Age (years)	1.020 (0.929–1.120)	0.683	0.985 (0.889–1.092)	0.776
BMI (kg/m^2^)	1.071 (0.980–1.171)	0.128	1.071 (0.975–1.177)	0.151

OR: Odds Ratio, CI: Confidence Interval. Univariate and multivariate logistic regression analyses were performed. * *p* < 0.05.

## Data Availability

The study datasets are available from the corresponding author on reasonable request, within the constraints of institutional data-sharing regulations and patient confidentiality.
